# Effects of the Th2-dominant milieu on allergic responses in Der f 1-activated mouse basophils and mast cells

**DOI:** 10.1038/s41598-018-25741-w

**Published:** 2018-05-16

**Authors:** Myung-hee Yi, Hyoung-Pyo Kim, Kyoung Yong Jeong, Ju Yeong Kim, In-Yong Lee, Tai-Soon Yong

**Affiliations:** 1 Department of Environmental Medical Biology, Arthropods of Medical Importance Resource Bank, Institute of Tropical Medicine, Yonsei University College of Medicine, Seoul, 03722 Korea; 2 Graduate Program of Nano Science and Technology, Yonsei University College of Medicine, Seoul, 03722 Korea; 3 Severance Biomedical Science Institute, Yonsei University College of Medicine, Seoul, 03722 Korea; 4 BK21 PLUS Project for Medical Science, Yonsei University College of Medicine, Seoul, 03722 Korea; 5 Department of Internal Medicine, Institute of Allergy, Yonsei University College of Medicine, Seoul, Korea

## Abstract

Although basophils and mast cells share similar phenotypic and functional properties, little is known about the difference in the initial Th2 immune responses of these cells following exposure to proteolytic allergens. Here, we investigated the mechanisms of Th2-mediated immune responses in mouse bone marrow-derived basophils (BMBs) and mast cells (BMMCs) via stimulation with the cysteine protease allergen Der f 1. Our results showed that Th2 cytokines were induced from BMBs by active recombinant Der f 1 (rDer f 1 independently with Toll-like receptor (TLR) 2 and TLR4. Although both BMBs and BMMCs expressed protease-activated receptors on their surfaces, PAR expression following exposure to rDer f 1 was altered only in basophils. G protein-coupled receptors in basophils were found to be associated with interleukin (IL)-4 and IL-13 production from BMBs upon Der f 1 treatment. Secretion of Th2 cytokines from rDer f 1-treated basophils was mediated by G protein βγ and phosphatidylinositol 3-kinase γ through the extracellular signal-regulated kinase and c-Jun N-terminal kinase pathways. These findings provide insights into the roles of cysteine proteases in Th2 immune responses, such as allergic diseases, and improve our understanding of the mechanisms of Th2 cytokine production.

## Introduction

T helper 2 immune responses are initiated by activation of primary effector cells, such as eosinophils, mast cells, and basophils. These cells contribute to host defence responses against parasites and play roles in the pro-inflammatory environment in response to allergens^1^. Basophils are rarely found in normal tissue; however, they accumulate in sites of inflammation^2^, where they facilitate secretion of histamine, leukotrienes, and cytokines^3^. Several studies have demonstrated that basophils are pivotal for Th2 immune responses and rapid generation of interleukin (IL)-4 and IL-13 in both humans and mice^4,5^. In addition, basophil-derived IL-4 has been shown to differentiate naïve CD4 T cells into Th2 effector cells, which play key roles in eliciting and maintaining allergic responses^6,7^. Basophils and mast cells may be derived from common precursor cells, which share similar phenotypic and functional characteristics. For example, these cells express FcεRI on their surfaces, and both cells are central effectors in Th2 immune reactions^8^. Despite sharing a number of phenotypic and functional properties, basophils and mast cells exhibit different activities. Although they play roles in allergic reactions, mast cells are known as the major effector cells in the immediate hypersensitivity reaction, whereas basophils are recruited to inflammatory sites, where they act to enhance the inflammatory process and thus may be associated with the severity of allergic diseases^9–12^.

A previous study reported that proteases from helminths and allergens induce the expression of type 2 cytokines from basophils^13^. Sokol *et al*. suggested that basophils are directly targeted by cysteine protease allergens in the absence of IgE-allergen complexes, thereby inducing the production of Th2-promoting cytokines and causing Th2 differentiation *in vivo*^14^. These data imply that the proteolytic activity of allergens is critical for activation of basophils before crosslinking of the FcεRI and IgE-allergen complex. However, the mechanisms of innate immune recognition of basophils associated with initiation of Th2-biased inflammatory responses via receptors other than FcεRI have not been investigated in detail. Furthermore, except for FcεRI, differences in the immune sensing mechanism of allergen recognition in basophils and mast cells are currently unknown.

In this study, we examined differences in Th2 cytokine production from mouse bone marrow-derived basophils (BMBs) and mast cells (BMMCs) in response to the cysteine protease allergen rDer f 1. Moreover, we investigated the effects of rDer f 1 on early immune changes associated with allergic reactions. Our results demonstrated that cysteine protease activity was important for the activation of BMBs and innate immune reactions, which were initiated by activation of G protein coupled receptors (GPCRs) and protease-activated receptors (PARs) in basophils but not mast cells.

## Results

### Production of IL-4 and IL-13 by proteolytically active Der f 1 in BMBs, not in BMMCs

**Figure 1:**
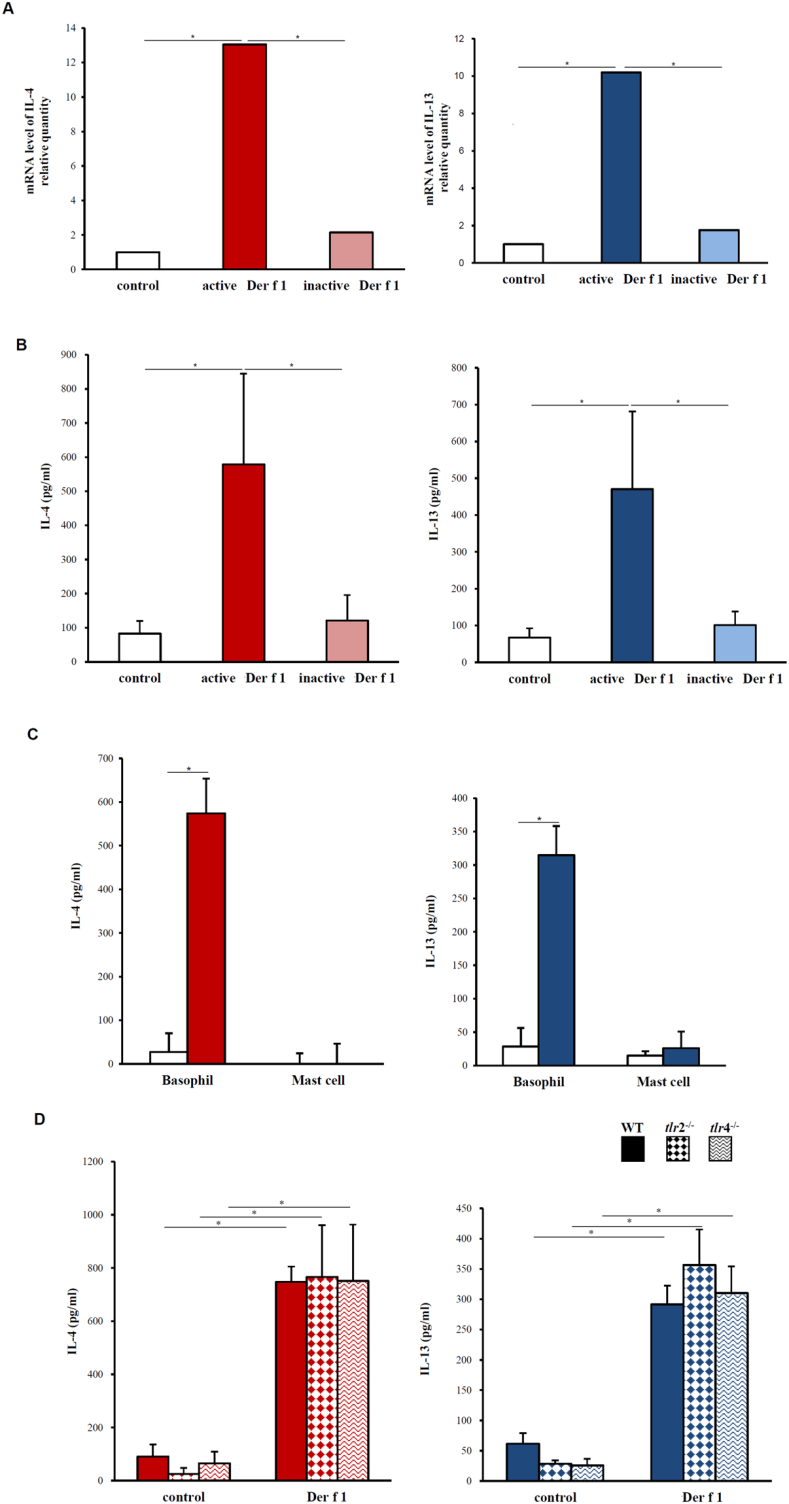
Production of IL-4 and IL-13 in BMBs and BMMCs upon Der f 1 stimulation. (**A**) Quantitative PCR of *IL-4* and *IL-13* transcript expression in BMBs 2 h after stimulation. (**B**) IL-4 and IL-13 secretion after stimulation of mouse BMBs with Der f 1 for 24 h. (**C**) IL-4 and IL-13 secretion after stimulation of BMBs and BMMCs with Der f 1. Data are presented as the mean ± SD of at least five independent experiments (*p < 0.05). (**D**) Der f 1-induced IL-4 and IL-13 secretion by BMBs from TLR2- or TLR4-knockout mice. Data are presented as the mean ± SD of at least three independent experiments (*p < 0.05).

To determine whether Der f 1 could induce Th2 cytokines from mouse BMBs and BMMC *in vitro*, we sorted out basophils and mast cells from mouse bone marrow cultures by flow cytometry to a purity of over 97%. In BMBs treated with proteolytically active Der f 1, expression of transcripts encoding of IL-4 and IL-13 was increased compared with that in unstimulated cells (Fig. 1A). Secretion of IL-4 and IL-13 in BMBs was confirmed by ELISA after stimulation with Der f 1 for 24 h (Fig. 1B). However, these cytokines were not expressed in BMMCs under the same conditions (Fig. 1C). In addition, the same results were obtained in bone marrow derived mucosal-like mast cells and connective tissue-like mast cells (Supplementary Figure S1). We also found that inactivated Der f 1 would not activate BMBs, indicating that proteolytic activity of allergens played a role in basophil activation (Fig. 1A,B). Taken together, these data showed that proteolytically active Der f 1 directly activated and induced Th2 cytokines independently of IgE antibody recognition of allergens on BMBs, but not BMMCs. In addition, these data implied that the proteolytic activity of allergens was critical for activation of some specific receptors on basophils.

### Th2 cytokine secretion in BMBs by Der f 1 via TLR2- or TLR4-independent pathways

Since pattern recognition receptors are mainly responsible for induction of innate immune reactions, we examined whether production of Th2 cytokines in basophils was affected by TLR2 and TLR4. Specifically, we measured the secretion of cytokines from Der f 1-activated BMBs in TLR2- and TLR4-deficient mice. The levels of Der f 1-induced IL-4 and IL-13 secretion in BMBs from TLR2- or TLR4-deficient mice were comparable to those of wild-type mice (Fig. 1D). These results indicated that Der f 1-induced IL-4 secretion was not affected by TLR2 or TLR4 (Fig. 1D). Similarly, IL-13 secretion was not affected by TLR2 or TLR 4 (Fig. 1D). Therefore, we concluded that Th2 cytokine production induced by Der f 1 was not associated with TLR2 or TLR4.

### PAR expression in BMBs and BMMCs

**Figure 2:**
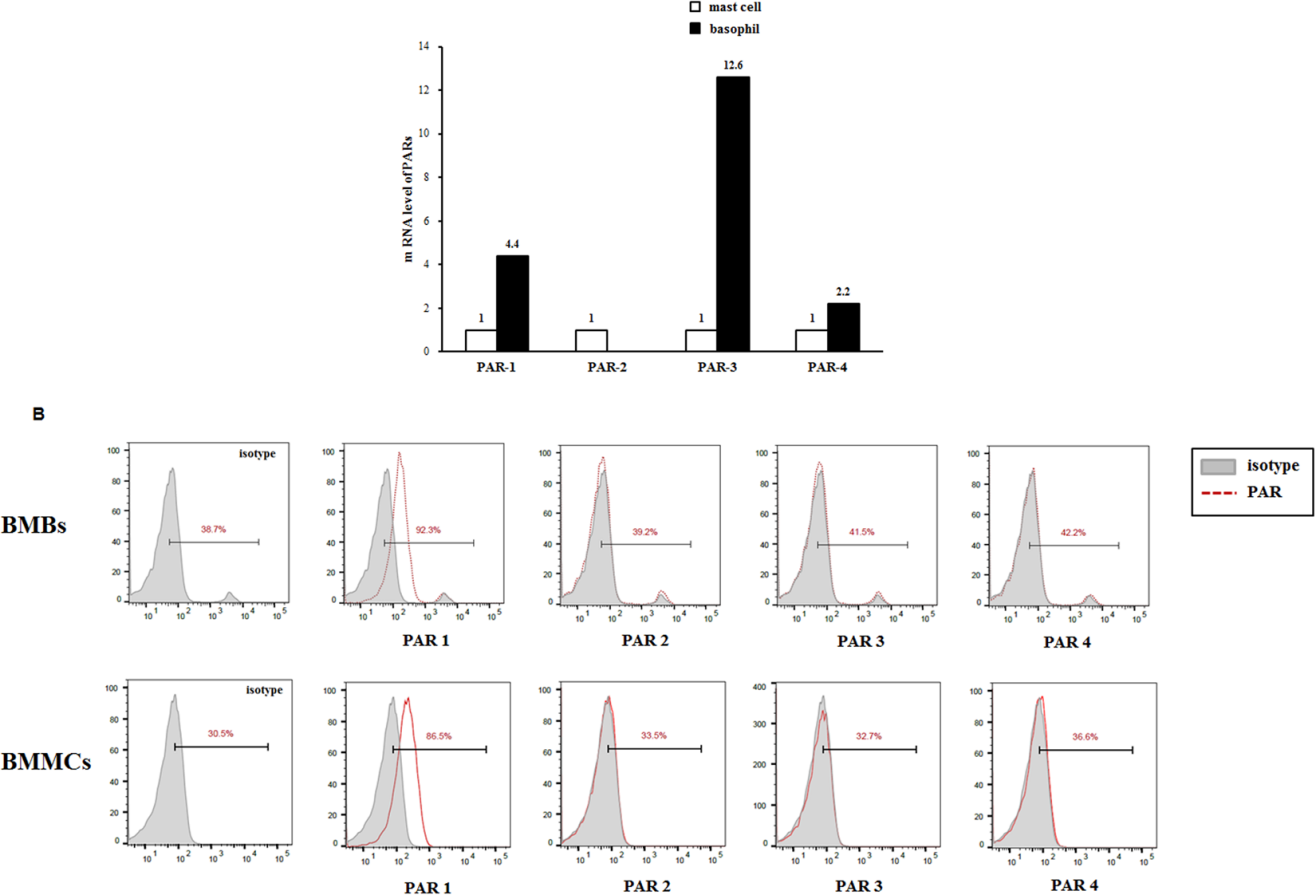
Expression of PARs on BMBs and BMMCs. (**A**) *PAR* mRNA levels in BMBs and BMMCs evaluated by real-time PCR Data are presented as the mean ± SD of at least three independent experiments (*p < 0.05). (**B**) The surface expression levels of PARs on BMBs (upper) and BMMCs (lower) in mouse bone marrow cultures were detected using flow cytometry. Data are representative of at least five independent experiments.

Human basophils do not express PARs^15^; however, it is unclear whether mouse basophils express surface PARs. Therefore, we first examined the expression *PAR-1*, *PAR-2*, *PAR-3*, and *PAR-4* mRNA in sorted BMBs and BMMCs using real-time PCR. *PAR-1* and *PAR-4* mRNA levels were upregulated in BMBs compared with those in BMMCs. Moreover, *PAR-3* mRNA levels in BMBs were increased at least 10-fold compared with *PAR-3* mRNA levels in BMMCs (Fig. 2A). However, mRNA expression of *PAR-2* was not detected. We also evaluated the surface expression levels of PARs on BMBs and BMMCs using flow cytometry. PAR-1 was expressed on mouse basophils and mast cells (Fig. 2B); however, surface expression of PAR-3 and PAR-4 was rarely observed on either BMBs or BMMCs (Fig. 2B).

### Change in PAR-1 and PAR-3 content in BMBs by Der f 1

**Figure 3:**
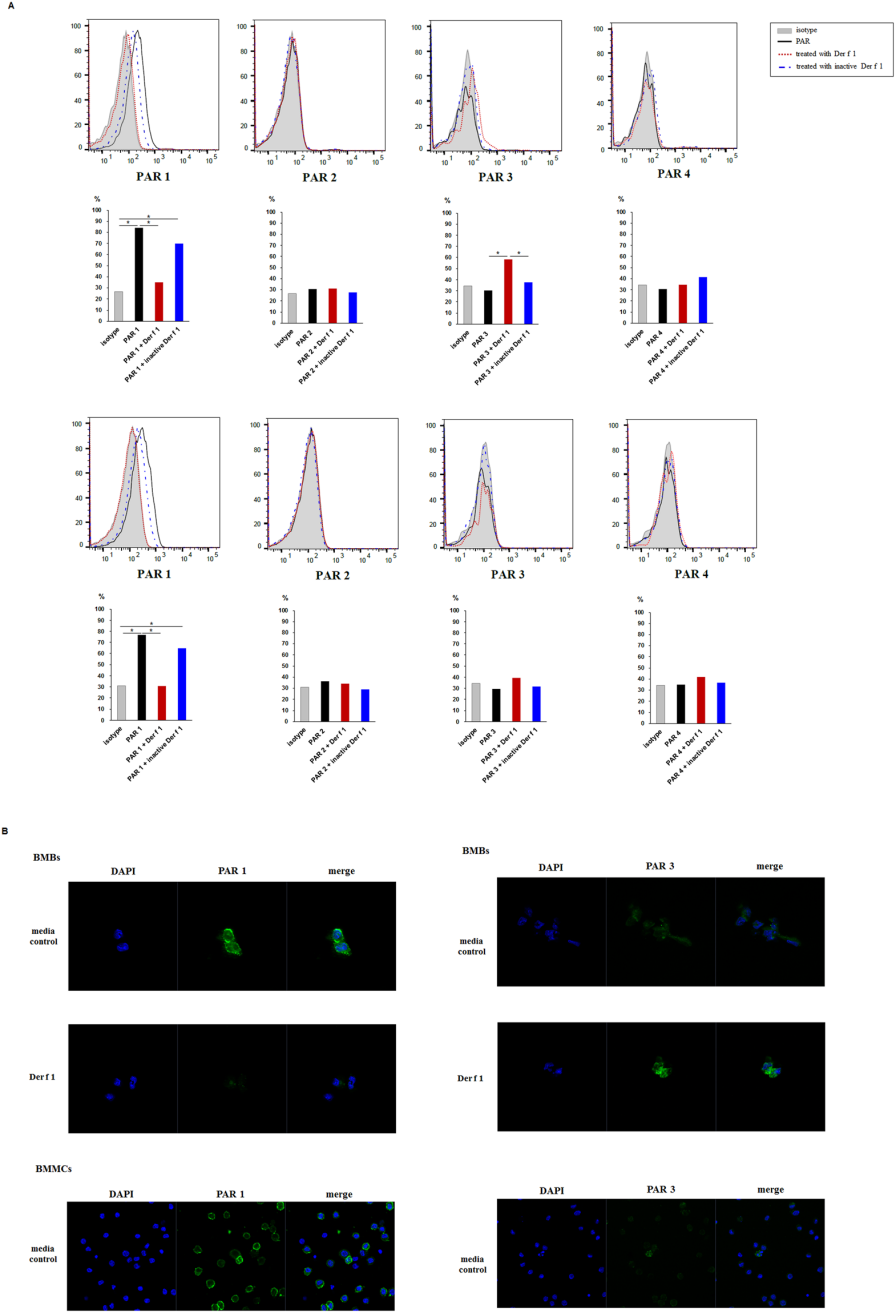
Expression of PARs on BMBs and BMMCs following treatment with Der f 1 or inactive Der f 1. (**A**) Expression of PARs on BMBs (upper) and BMMCs (lower) following treatment with Der f 1 and inactive Der f 1 at 1 h was detected by flow cytometry. Data are presented as the mean ± SD of at six independent experiments. (**B**) Expression of PAR-1 (left) and PAR-3 (right) on BMBs (upper) and BMMCs (lower) following treatment with Der f 1 was detected by confocal laser scanning microscopy. Results are representative of at five independent experiments.

We assumed that PARs could be activated by Der f 1 to produce Th2 cytokines because Der f 1 is cysteine protease. To investigate whether Der f 1 influenced PAR expression levels in BMBs and BMMCs, we measured surface expression of PARs expressed on BMBs and BMMCs after stimulation with Der f 1 for 1 h. Interestingly, Der f 1 treatment decreased the levels of PAR-1 on BMBs and BMMCs. As a control, treatment with inactive Der f 1 did not change PAR-1 content in BMBs and BMMCs (Fig. 3A). However, the PAR-3 content of BMBs increased upon treatment with Der f 1, whereas PAR-3 expression was not changed upon treatment with heat-inactivated Der f 1 (Fig. 3A). In contrast, PAR-3 expression on BMMCs was not detected after treatment with Der f 1 under the same conditions (Fig. 3A).

We next confirmed the expression levels of PARs on sorted BMBs and BMMCs using confocal microscopy. PAR-1 was expressed on BMBs and BMMCs (Fig. 3B). PAR-1 expression on BMBs and BMMCs decreased after treatment with Der f 1 (Fig. 3B). Interestingly, PAR-3 was also expressed on sorted BMBs, and its expression was enhanced upon treatment with Der f 1 (Fig. 3B), whereas PAR-3 content on the surface of BMMCs did not change, even after Der f 1 treatment (Fig. 3B). Taken together, these data indicated that PAR-1 at the surface of BMBs and BMMCs was decreased via the proteolytic activity of Der f 1. The expression of PAR-3 was increased by Der f 1 only on mouse basophils, but not on mast cells.

### Secretion of IL-4 and IL-13 in BMBs activated by Der f 1 was independent of PAR-1

**Figure 4:**
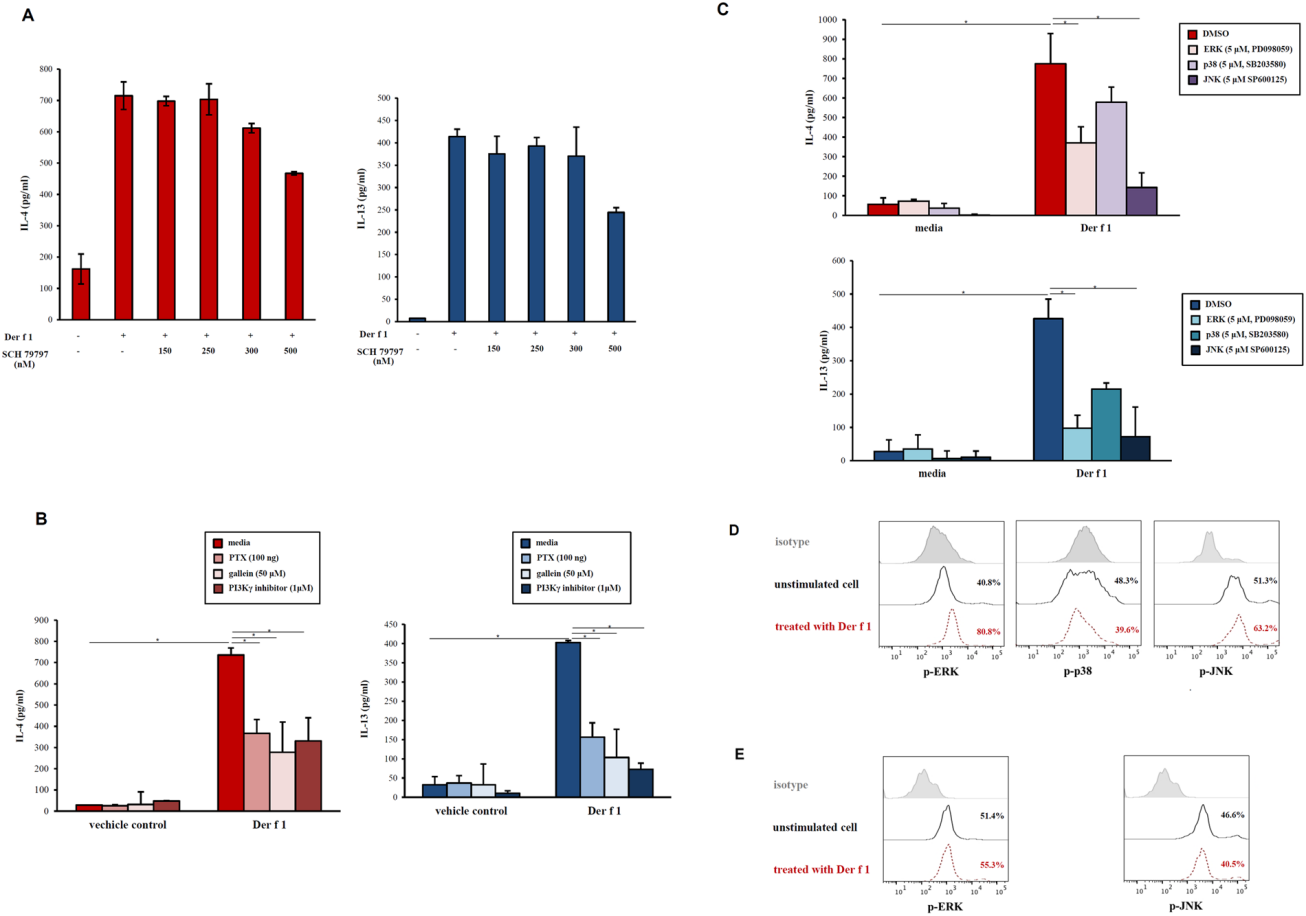
Effects of PAR-1, GPCR, PI3K, and Gβγ proteins and MAPK inhibitors on Der f 1-induced IL-4 and IL-13 production in BMBs. (**A**) Effects of PAR1 on Der f 1-induced IL-4 and IL-13 production in BMBs. BMBs were pretreated with pharmacological inhibitors SCH79797 (PAR-1 antagonist, ~500 nM) and then activated with Der f 1. Data are presented as the mean ± SD of at least three independent experiments. (**B**) BMBs were pretreated with PTX (GPCR inhibitor), PI3Kγ inhibitor, or gallein (Gβγ inhibitor), followed by activation with Der f 1. Data are presented as the mean ± SD of at least three independent experiments (*p < 0.05). (**C**) BMBs were pretreated with the pharmacological inhibitors PD098059 (ERK inhibitor), SB203580 (p38 inhibitor), and SP600125 (JNK inhibitor), followed by activation with Der f 1. Data are presented as the mean ± SD of at least five independent experiments (*p < 0.05). (**D**) ERK, p38, and JNK phosphorylation levels were analyzed by flow cytometry in BMBs stimulated with Der f 1 for 10 min. Data are representative of three independent experiments. (**E**) Effects of Der f 1 on ERK and JNK phosphorylation in BMMCs. BMMCs were stimulated with Der f 1 for 10 min and then evaluated by flow cytometry. Data are representative of three independent experiments.

To investigate whether PAR-1 was involved in IL-4 and IL-13 production by Der f 1-activated BMBs, we treated BMBs with various concentrations of a PAR-1 antagonist. The PAR-1 antagonist had no effect on the production of IL-4 and IL-13 by BMBs (Fig. 4A). These results indicated that PAR-1 was not directly involved in the secretion of IL-4 and IL-13 from BMBs *in vitro*.

### Role of GPCRs in Der f 1-induced secretion of IL-4 and IL-13 from BMBs

Because PAR-1 was not directly associated with the production of Th2 cytokines and PAR-3 did not regulate cellular signalling pathways, we next asked whether GPCRs, which include PARs, played a role in IL-4 and IL-13 secretion upon treatment with Der f 1. BMBs were pretreated with PTX, which is known to block the coupling and activation of G proteins, followed by treatment with Der f 1. Der f 1-induced IL-4 secretion in basophils decreased dramatically following treatment with PTX (Fig. 4B), and IL-13 secretion was also inhibited by PTX (Fig. 4B). These results suggested that GPCRs were involved in secretion of Th2 cytokines such as IL-4 and IL-13, by the cysteine protease Der f 1.

### Expression of IL-4 and IL-13 in BMBs upon Der f 1 treatment involved Gβγ and PI3K

In order to investigate the possible roles of GPCRs in the regulation of Der f 1, several molecules involved in GPCR signalling were examined in Der f 1-activated basophils using an inhibitor of G protein βγ (gallein). We also evaluated whether Gβγ-activated PI3Kγ was involved in Der f 1-induced Th2 cytokine production. IL-4 and IL-13 production was significantly decreased upon Der f 1 treatment in the presence of specific inhibitors of Gβγ and PI3Kγ (Fig. 4B). These data indicated that GPCRs were involved in the secretion of Th2 cytokines through a signalling cascade involving Gβγ and PI3Kγ, although the exact GPCR was not identified.

### Production of IL-4 and IL-13 was dependent on ERK and JNK

Because GPCRs coupled to heterotrimeric G proteins can stimulate MAPKs through Gβγ, we next examined whether the MAPK pathway was involved in Der f 1-induced Th2 cytokine production. The effects of pharmacological inhibitors of ERK, p38, and JNK were evaluated in Der f 1-activated BMBs. ERK and JNK inhibitors reduced IL-4 and IL-13 secretion, whereas the p38 inhibitor did not (Fig. 4C). These data indicated that ERK and JNK pathways regulated Der f 1-induced Th2 cytokine secretion. In addition, we confirmed phosphorylation of MAPKs using flow cytometry. Der f 1 increased the levels of phosphorylated ERK and JNK but not p38 MAPK (Fig. 4D). However, Der f 1 was not able to induce ERK and JNK phosphorylation in mouse BMMCs (Fig. 4E). These data indicated that activation of ERK and JNK was an important step for Der f 1-induced Th2 cytokine secretion from basophils.

## Discussion

Basophils and mast cells are critical regulators of inflammatory reactions by FcεRI and IgE-mediated crosslinking to induce initial production of Th2 cytokines^4,6,7^. In this study, we showed that Der f 1, which exhibits cysteine protease activity, can directly activate mouse basophils but not mast cells to secrete type 2 cytokines before IgE-sensitization. Basophils are important cells that facilitate Th2 effector cell differentiation of naïve T cells through production of IL-4^7^. Basophils have been shown to initiate Th2 differentiation of naïve T cells in ovalbumin-induced allergic airway inflammation by inducing the expression of IL-4^16^. These data suggest that basophils may possess the potential to induce the development of Th2-type immune responses. In our study, BMBs, not BMMCs, were shown to express high mRNA and protein levels of IL-4 and IL-13 after stimulation with the cysteine protease allergen Der f 1. In addition, our results implied that some particular receptors were expressed on basophils following activation of Der f 1.

The contributions of TLR2 and TLR4 to house dust mite-induced allergic inflammatory responses have been described previously. Group 2 mite allergens (Der p 2 and Der f 2) and lipopolysaccharide (LPS)-binding proteins are known to mimic MD2, a TLR4 coreceptor, both structurally and functionally, thereby promoting airway inflammation in a TLR4-dependent manner upon exposure to very low concentrations of LPS^17–19^. However, secretion of IL-4 and IL-13 from BMBs upon Der f 1 activation did not appear to be affected by the presence of TLR2 nor TLR4. In addition, this finding indicated that the production of Th2 cytokines in BMBs activated by Der f 1 could not be attributed to the activation of microbial products, such as β-glucans and LPS, the activities of which are mediated by TLR2 and TLR4, respectively.

Next, we attempted to identify the receptor responsible for the initiation of immune responses and Th2 cytokine production upon protease allergen exposure. Since PARs are cleaved and activated by certain proteases, we assumed that PARs could be candidate receptors of BMBs. Human basophils from peripheral blood do not express PARs^15^; however, no studies had yet examined whether PARs are expressed in mouse basophils. In this study, we demonstrated expression of *PAR-1*, *PAR-3*, and *PAR-4* mRNAs in BMBs and showed that PAR-1 and PAR-3 were expressed on the cellular membrane of BMBs by flow cytometry and confocal microscopy. Interestingly, PAR-3 expression on the surface of BMBs increased slightly following exposure to the protease allergen Der f 1, whereas PAR-3 expression on BMMCs was not detected under the same conditions. Although it is still unclear whether increased expression of PAR-3 on basophils upon Der f 1 treatment is involved in production of Th2 cytokines, we can speculate that enhanced expression of PAR-3 may support the activation of PAR-1 or PAR-4 by dimerization or polymerization through the so-called “unique cofactoring mechanism”^20,21^ or somehow influences inflammatory reactions in response to other proteases. We also found that PAR-1 protein content on BMBs and BMMCs decreased following treatment with proteolytically active Der f 1. However, when we utilized a selective antagonist of PAR-1 (SCH79797), which binds to the PAR-1 recognition motif and inhibits the binding of the tethered-ligand complex, we found that SCH79797 did not affect the secretion of IL-4 and IL-13 from Der f 1-activated BMBs. Furthermore, BMMCs did not secrete Th2 cytokines when treated with Der f 1, despite the decrease in PAR1 expression. Taken together, these results showed that there was no direct association of PAR-1 with activation of BMBs or BMMCs *in vitro*.

Because PARs are not thought to be directly involved in the secretion of IL-4 and IL-13, we next examined the role of GPCRs. Interestingly, secretion of IL-4 and IL-13 induced by Der f 1 in BMBs decreased dramatically following treatment with PTX, indicating that GPCRs may affect the production of IL-4 and IL-13 in BMBs. GPCRs are coupled to heterotrimeric G proteins (Gα, Gβ, and Gγ), and Gβγ complex-dependent signalling is known to modulate inflammatory and immune responses through a signalling cascade^22,23^. In addition, a previous study suggested that Gβγ activation may regulate the induction of Th2 cytokines, such as IL-13 via ERK1/2 activation, thereby mediating airway hyper-responsiveness and inflammation^24^. The results of this study supported the possibility that Gβγ protein-dependent signalling may be associated with Th2 cytokine expression in BMBs induced by Der f 1 exposure. Moreover, our results suggested that PI3Kγ was affected by the Gβγ complex, which could relay intracellular signals in BMBs to facilitate the secretion of Th2 cytokines. These findings are consistent with previous observations showing that PI3Kγ is specifically activated by Gβγ complexes in leukocytes^25,26^. Furthermore, we showed that Der f 1 activated ERK and JNK phosphorylation in parallel and that chemical inhibitors of ERK and JNK, but not p38 MAPK, inhibited IL-4 and IL-13 production. A previous study suggested calcium release-activated channels, including nuclear factor of activated T-cell signalling, play important roles in secretion of Th2 cytokines in basophils through papain, a cysteine protease, and that the involved signalling pathways are not different from those of mast cells^27,28^. In contrast, Der f 1 did not induce phosphorylation of MAPKs or secretion of IL-4 and IL-13 in BMMCs, indicating that mast cells could not be activated by Der f 1.

Based on the results of this study, we concluded that basophils, not mast cells, were directly activated by the cysteine protease allergen Der f 1 prior to IgE sensitization. In addition, we showed that basophils produced Th2 cytokines, including IL-4 and IL-13, through TLR2- and TLR4-independent pathways and through an IgE-independent pathway. A basophil-derived cytokine milieu promotes differentiation of activated naïve CD4 T cells towards Th2-type effector T cells and suppresses Th1-type immune responses^7^. In this way, our data revealed that basophils may elicit a Th2-dominant cytokine milieu upon Der f 1 stimulation, thereby playing a regulatory role in determining the fate of naïve T cells. Importantly, this phenomenon may explain why Th2-biased immune responses to parasite infections are highly associated with basophils that produce IL-4 upon exposure to cysteine proteases secreted from helminths. This study is the first demonstration that mouse basophils express PARs (PAR-1, -2, and -4) and that PAR-1 and -3 expression on the surface of BMBs could be regulated by Der f 1. However, further studies are needed to better understand the initial mechanism of the Th2 immune response, particularly with regard to how PARs are activated by proteolytic allergens in basophils, signalling pathways including those of GPCRs, and the role of protease-activated basophils under inflammatory conditions. Elucidation of the mechanism of basophil activation by protease allergens before IgE sensitization may shed light on the initial stages of allergic reactions and facilitate development of new therapeutic approaches. In addition, further in-depth studies are needed to elucidate the responses of mast cells and basophils to allergens and their cellular signalling pathways.

## Materials and Methods

### Production of recombinant Der f 1

Recombinant Der f 1 was produced in *Pichia* as described previously^29,30^. Briefly, recombinant protein secreted as a proform of Der f 1 with 6× His at the C-terminus was harvested and purified using Ni-affinity chromatography. The mature form of Der f 1 with protease activity was obtained by dialysis against 100 mM acetate buffer.

### Antibodies and reagents

Phycoerythrin (PE)-conjugated anti-mouse FcεRIα (MAR-1) and fluorescein isothiocyanate (FITC)-conjugated anti-mouse c-Kit/CD117 (2B8) were purchased from eBioscience (San Diego, CA, USA). PE-Cy7 anti-mouse CD49 (DX5) was from BioLegend (San Diego, CA, USA). Purified 2.4G2 antibodies targeting the Fc receptor (Mouse Fc block), Alexa Fluor 647 mouse anti-extracellular signal-regulated kinase (ERK) 1/2 (pT202/pY204), Alexa Fluor 647 mouse anti-p38 mitogen-activated protein kinase (MAPK; pT180/pY182), Alexa Fluor 647 mouse anti-c-Jun N-terminal kinase (JNK; pT183/pY185) and Alexa Fluor 647 mouse IgG1κ isotype control were purchased from BD Pharmingen (San Diego, CA, USA). The following inhibitors were purchased from Calbiochem (La Jolla, CA, USA): MAPK/ERK inhibitor (PD98059), p38 inhibitor (SB203580), JNK inhibitor II (SP600125), phosphatidylinositol 3-kinase γ (PI3Kγ) inhibitor, and G protein βγ inhibitor (gallein). *Pertussis* toxin (PTX) was obtained from Sigma-Aldrich (St. Louis, MO, USA), and anti-goat thrombin R (S-19; protease-activated receptor [PAR]-1), PAR-2 (S-19), PAR-3 (M-14), PAR-4 (S-20), normal goat IgG antibodies, and donkey anti-goat IgG-PerCP secondary antibodies for PAR-1-4 were purchased from Santa Cruz Biotechnology Inc. (Dallas, TX, USA). The PAR-1 antagonist SCH 79797 and histamine receptor 4 (H4R) antagonist JNJ-7777120 were obtained from Axon Medchem (Reston, VA, USA).

### Mice

BALB/c mice were purchased from Orient Bio (Seongnam, Korea). Toll-like receptor (TLR) 2^−/−^ and TLR4^−/−^ BALB/c mice were provided by Prof. Ji-Hwan Ryu (Research Center for Human Natural Defense System, Yonsei University, Seoul, Korea). All animal studies were approved by the Department of Laboratory Animal Resources Committee of Yonsei University College of Medicine (No. 2014–0086, 2016–0230).

### Bone marrow culture and sorting

For the production of BMBs and BMMCs, erythrocyte-depleted mouse femoral bone marrow cells were cultured in RPMI 1640 (GenDEPOT, Barker, TX, USA) supplemented with 10% heat-inactivated fetal bovine serum, 100 U/mL penicillin/streptomycin (GenDEPOT), 50 μM 2-mercaptoethanol (Sigma-Aldrich), and 30 ng/mL IL-3 (PeproTech, Rocky Hill, NJ, USA) for 8 days. Cultured bone marrow cells were first blocked with 2.4G2 for elimination of Fc receptor-mediated antibody binding. Samples were sorted with a BD FACSAria III cell sorter (BD Biosciences, San Diego, CA, USA), and BMBs and BMMCs were selected based on CD49 + FcεRI + c-Kit– and CD49-FcεRI + c-Kit + staining, respectively. Both populations were sorted to a purity of greater than 98%.

### Real-time polymerase chain reaction (PCR)

RNA was isolated from cells using TRIzol reagent (Invitrogen Life Technologies, Carlsbad, CA, USA). cDNA was reverse transcribed from total RNA samples using a High-Capacity RNA-to-cDNA kit (Applied Biosystems, Foster City, CA, USA). Quantitative reverse transcription (RT)-PCR was performed by monitoring the synthesis of double stranded DNA during 40 PCR cycles with SYBR Green PCR Master Mix (Applied Biosystems). The sequences of the primers were as follows: glyceraldehyde-3-phosphate dehydrogenase (*GAPDH*), 5′-TGTGTCCGTCGTGGATCTGA-3′ (forward) and 5′-CCTGCTTCACCACCTTCTTGA-3′ (reverse); *IL-4*, 5′-TCGGCATTTTGAACGAGGTC-3′ (forward) and 5′-GAAAAGCCCGAAAGAGTCTC-3′ (reverse); *IL-13*, 5′-GACCAGACTCCCCTGTGCA-3′ (forward) and 5′-TGGGTCCTGTAGATGGCATTG-3′ (reverse); *PAR-1*, 5-CAGCCAGAATCAGAGAGGACAGA-3′ (forward) and 5′-CCAGCAGGACGCTTTCATTT-3′ (reverse); *PAR-2*, 5′-AGCCGGACCGAGAACCTT-3′ (forward) and 5′-GGAACCCCTTTCCCAGTGATT-3′ (reverse); *PAR-3*, 5′-GCTCCATTTTTCAGCTCCTC-3′ (forward) and 5′-GGAGGGAAGGGGACATGTAT-3′ (reverse); *PAR-4*, 5′-GCTACAGCCATGCACTCAGA-3′ (forward) and 5′-GCTACAGCCATGCACTCAGA-3′ and 5′-AGGGCTCGGGTTTGAATAGT-3′ (reverse). Relative mRNA levels were quantified using the ΔΔCt method and normalized to *GAPDH*. For each sample, triplicate reactions were analyzed.

### Enzyme-linked immunosorbent assay (ELISA)

For measurement of cytokine production, BMBs (2 × 10^5^/well) and BMMCs (2 × 10^5^/well) were seeded in 96-well tissue culture plates and incubated for the indicated times with or without rDer f 1 (100 μg) or heat-inactivated rDer f 1. The amount of cytokine production in medium was measured with Quantikine mouse IL-4 and IL-13 ELISA kits (R&D Systems, Minneapolis, MN, USA) according to the manufacturer’s instructions.

### Detection of PAR expression on BMBs and BMMCs by flow cytometry

For detection of PAR expression on BMBs and BMMCs, cultured bone marrow-derived cells (1 × 10^6^) were incubated with primary PAR-1, -2, -3, and -4 antibodies at a 1:200 dilution at room temperature, followed by incubation with donkey anti-goat IgG-PerCP secondary antibody (Santa Cruz Biotechnology Inc.) at a 1:1000 dilution for 1 h at 4 °C. Bone marrow-derived cells were gated using CD49^+^FcεRI^+^c-Kit^−^ and CD49^−^FcεRI^+^c-Kit^+^, which are markers of BMBs and BMMCs, respectively. To investigate the effects of the proteolytic activity of Der f 1 on the expression of PARs on BMBs and BMMCs, cells were treated with rDer f 1 for 1 h at 37 °C, after which PAR expression was estimated by flow cytometry using an LSRII instrument (BD Biosciences). All data were analyzed with FlowJo software (TreeStar, Ashland, OR, USA).

### Immunocytochemistry

BMBs and BMMCs were fixed and permeabilized by incubation in 4% paraformaldehyde and then methanol for 20 min at room temperature. Nonspecific binding sites were blocked by incubation with 0.2 mL phosphate-buffered saline containing 1% bovine serum albumin for 1 h at room temperature. Cells were stained by incubating with primary antibodies (1:200) and then treated with anti-goat IgG-FITC secondary antibodies. Fluorescence images were obtained with a Zeiss LSM 700 confocal microscope (Carl Zeiss, Berlin, Germany). All data were obtained from at least three independent experiments.

### Chemical exposure

To investigate the effects of G protein-coupled receptors (GPCRs) on Der f 1-induced Th2 cytokines, BMBs were pretreated with 100 ng PTX, followed by activation with Der f 1. To examine whether GPCR-related signal molecules were involved in basophil activation, BMBs were incubated with specific inhibitors of Gβγ (50 μΜ, gallein) and PI3K (1 μM, LY294002) for 30 min before treatment with Der f 1. To evaluate the effects of MAPKs on Der f 1-induced Th2 cytokine production, cells were treated with specific inhibitors for ERK (PD098059, 5 μM), p38 MAPK (SB203580, 5 μM), and JNK (SP600125, 5 μM) for 30 min before stimulation with Der f 1.

### Detection of MAPK phosphorylation by flow cytometry

Sorted BMBs and BMMCs were activated with Der f 1 and fixed using Lyse/Fix Buffer 5× (BD Biosciences) for detection of MAPK phosphorylation. After fixation, samples were resuspended in Perm Buffer III (BD Biosciences) on ice for 30 min. Cells were labelled with phospho-specific anti-ERK, anti-p38, and anti-JNK antibodies for 1 h at room temperature. Mouse IgG1κ was used as an isotype control. Intracellular phosphorylation in BMBs was measured by flow cytometry using an LSRII instrument (BD Biosciences).

### Statistical analyses

Results are presented as the mean ± standard deviation (SD) of at least three separate experiments. Statistical significance was determined using Student’s *t*-test with Sigma Plot 9.0. (San Jose, CA, USA). Differences with *p* values of 0.05 or less were considered statistically significant.

## Electronic supplementary material

supplementary data
