# Supplementary material for: Effects of the Th2-dominant milieu on allergic responses in Der f 1-activated mouse basophils and mast cells
Source: Sci Rep. 2018 May 16;8:7706. doi: 10.1038/s41598-018-25741-w (PMC5955989; doi:10.1038/s41598-018-25741-w)
Supplement: Supplementary file 1 — supplementary data [file 41598_2018_25741_MOESM1_ESM.pdf]

Effects of the Th2-dominant milieu on allergic responses in Der f 1-activated mouse basophils and mast cells

Myung-hee Yi[1], Hyoung-Pyo Kim[1], Kyoung Yong Jeong[2], Ju Yeong Kim[1], In-Yong Lee[1], and Tai-Soon Yong[1*]

[1]Department of Environmental Medical Biology, Arthropods of Medical Importance Resource Bank, Institute of Tropical Medicine and Yonsei University College of Medicine, Seoul, Korea

[2]Department of Internal Medicine, Institute of Allergy and Yonsei University College of Medicine, Seoul, Korea

*Corresponding author: Tai-Soon Yong, MD, PhD

Department of Environmental Medical Biology and Arthropods of Medical Importance Resource Bank, Institute of Tropical Medicine, Yonsei University College of Medicine, Yonsei-ro 50-1, Seodaemun-gu, Seoul 03722, Korea

Tel: 82-2-2228-1851

Fax: 82-2-363-8676

E-mail: tsyong212@yuhs.ac

Results

For comparison with the results obtained when BMMCs were stimulated by Der f 1, we differentiated mouse bone marrow stem cells into mucosal-like mast cells (MLMC) and connective tissue-like mast cells (CTLMC) using two different protocols (Supplementary reference 1). Secretion of IL-4 and IL-13 in MLMC and CTLMC was analysed by ELISA after stimulation with Der f 1 for 24 h. However, these cytokines were expressed in neither MLMC nor CTLMC.

Methods

Cell cultures

Mouse bone marrow cells were differentiated into cells with either a connective tissue-like phenotype (connective tissue-like mast cells, CTLMC) or a mucosal-like phenotype (mucosal-like mast cells, MLMC) as described previously[1]. Briefly, MLMC were obtained by culturing mouse bone marrow cells in DMEM (Gibco) containing 10% inactivated FBS (Gibco), 2 mM L-glutamine, 1 mM sodium pyruvate, 100 IU/mL penicillin G, 100 μg/mL streptomycin, and supplemented with either 50 ng/mL recombinant murine SCF, 20 ng/mL recombinant murine IL-3, 5 ng/mL recombinant murine IL-9, and 1 ng/mL recombinant human TGF-β1 (PeproTech). CTLMC were produced in RPMI 1640 medium (Gibco) containing 10% inactivated FBS, 4 mM L-glutamine, 1 mM sodium pyruvate, 100 IU/mL penicillin G, 100 μg/mL streptomycin (Gibco), 0.1 mM MEM nonessential amino acids (Gibco), and 50 μM 2-ME (Gibco), supplemented with either 50 ng/mL recombinant murine SCF and 1 ng/mL murine IL-4 (PeproTech). All cells were cultured for 3 weeks and the maturity and purity of the cells were examined by flow cytometric analysis for the expression of c-kit and FcεRI, using FITC-anti-mouse CD117 (c-kit) mAb 2B8 and PE-conjugated anti-

mouse FcεRI mAb MAR-1 (BioLegend). MLMC (2×10^5/well) and CTLMC (2×10^5/well) were seeded in 96-well tissue culture plates and activated with Der f 1 (100 µg) for 24 h. The amount of cytokine produced in the medium was measured with Quantikine mouse IL-4 and IL-13 ELISA kits (R&D Systems).

References

1. Ekoff, M., Strasser, A., Nilsson, G. FcεRI aggregation promotes survival of connective tissue-like mast cells but not mucosal-like mast cells. *J. Immunol.* **178**, 4177-4183 (2007).

Figure Legends

SUPPLEMENTARY FIGURE S1. Secretion of IL-4 and IL-13 in bone marrow derived MLMC (**A**) and CTLMC (**B**) upon Der f 1 stimulation. Data are presented as the mean ± SD of at least three independent experiments.

A

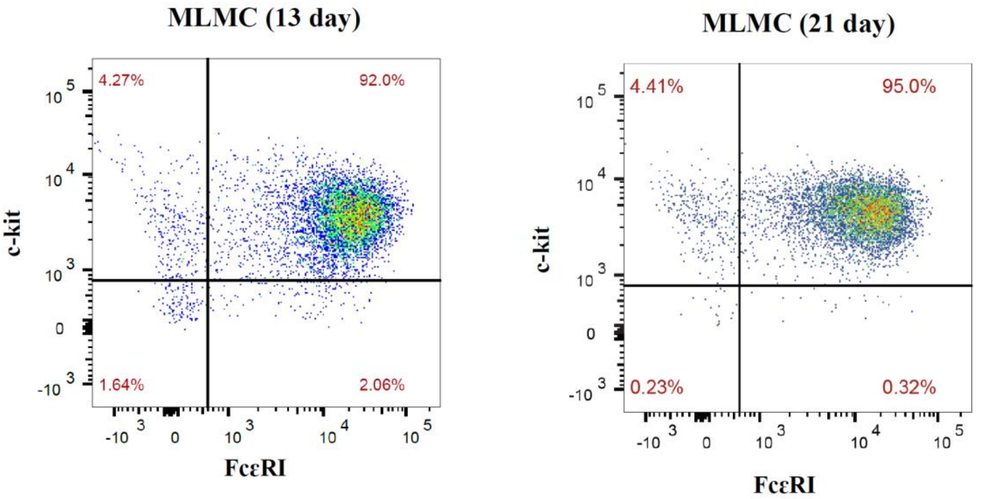
MLMC (13 day)
4.27%
92.0%
1.64%
2.06%
c-kit
FcεRI
MLMC (21 day)
4.41%
95.0%
0.23%
0.32%
c-kit
FcεRI

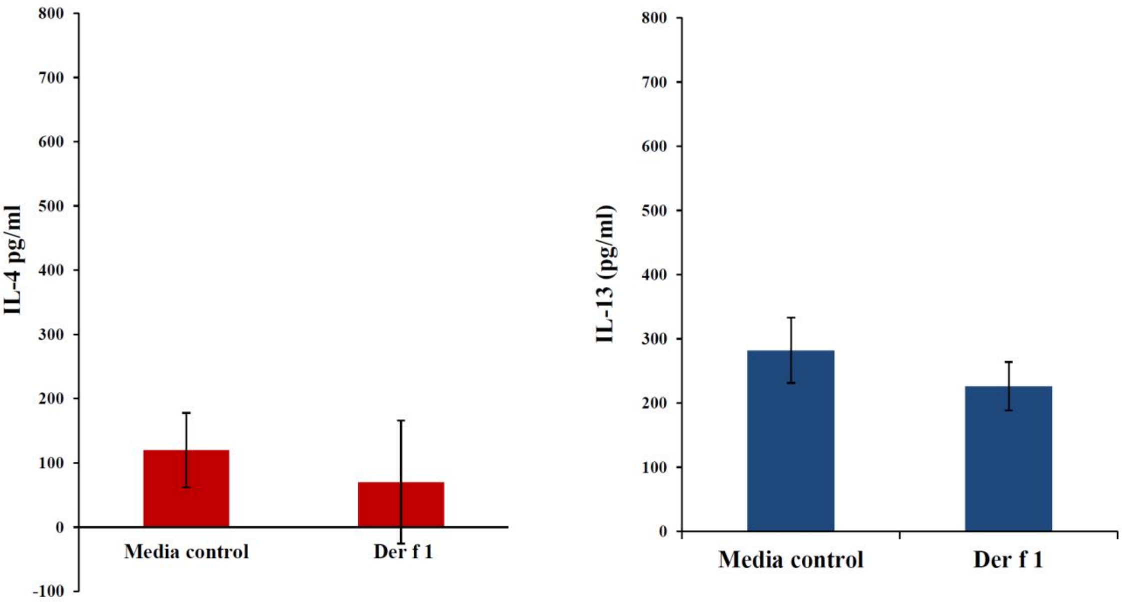
IL-4 pg/ml
Media control
Der f 1
IL-13 (pg/ml)
Media control
Der f 1

B

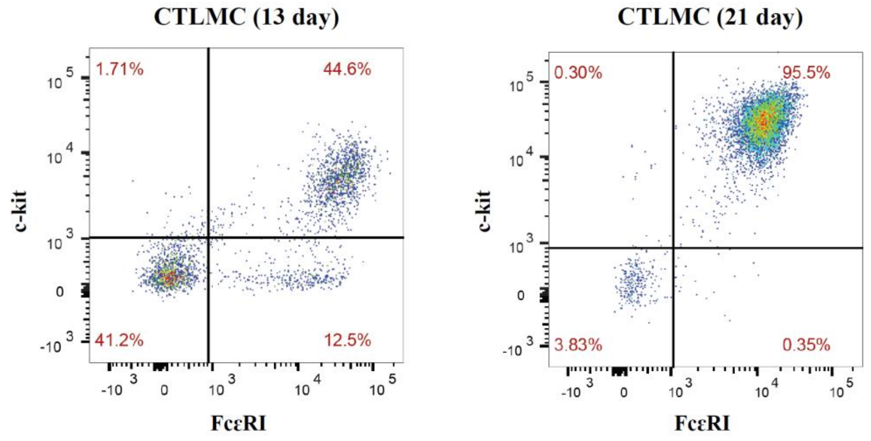

CTLMC (13 day)
1.71%
44.6%
41.2%
12.5%
c-kit
FcεRI
CTLMC (21 day)
0.30%
95.5%
3.83%
0.35%
c-kit
FcεRI

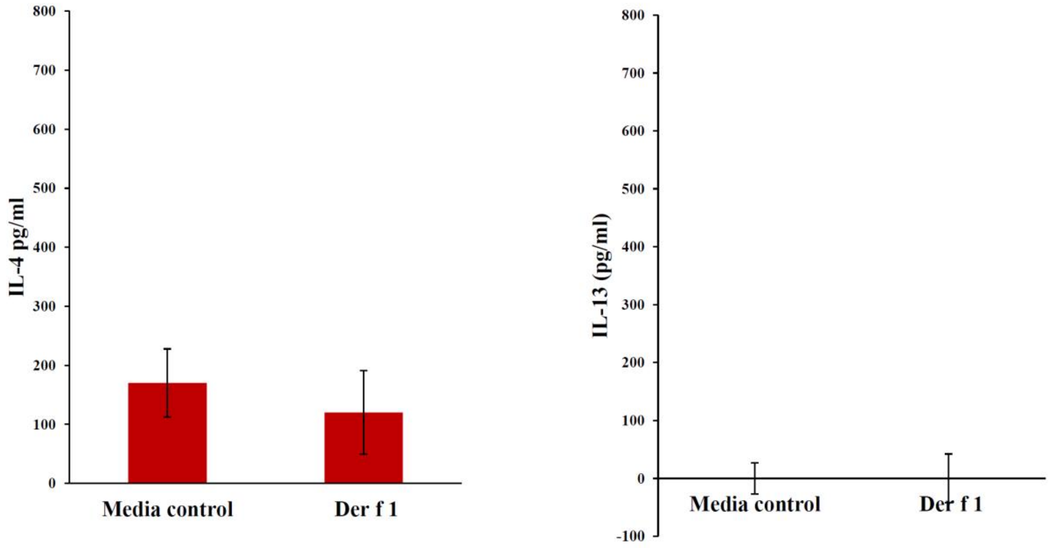

IL-4 pg/ml
Media control
Der f 1
IL-13 (pg/ml)
Media control
Der f 1
